# Application of enhanced assimilable organic carbon method across operational drinking water systems

**DOI:** 10.1371/journal.pone.0225477

**Published:** 2019-12-06

**Authors:** Frances C. Pick, Katherine E. Fish, Catherine A. Biggs, Jonathan P. Moses, Graeme Moore, Joby B. Boxall

**Affiliations:** 1 Pennine Water Group, Department of Civil and Structural Engineering, The University of Sheffield, Sheffield, United Kingdom; 2 Scottish Water, Perth, United Kingdom; 3 School of Engineering, Newcastle University, Newcastle, United Kingdom; 4 Scottish Water, Edinburgh, United Kingdom; Purdue University, UNITED STATES

## Abstract

Assimilable organic carbon (AOC) is known to correlate with microbial growth, which can consequently degrade drinking water quality. Despite this, there is no standardised AOC test that can be applied to drinking water distribution systems (DWDS). Herein we report the development of a quick, robust AOC that incorporates known strains *Pseudomonas fluorescens* strain P-17 and *Spirillum* strain NOX, a higher inoculum volume and enumeration using flow cytometry to generate a quicker (total test time reduced from 14 to 8 days), robust method. We apply the developed AOC test to twenty drinking water treatment works (WTW) to validate the method reproducibility and resolution across a wide range of AOC concentrations. Subsequently, AOC was quantified at 32 sample points, over four DWDS, for a year in order to identify sinks and sources of AOC in operative networks. Application of the developed AOC protocol provided a previously unavailable insight and novel evidence of pipes and service reservoirs exhibiting different AOC and regrowth behaviour. Observed correlations between AOC and microbial growth highlight the importance of monitoring AOC as an integral part of managing drinking water quality at the consumers tap.

## Introduction

Water utilities aim to produce biologically stable water to prevent the microbial status of drinking water deteriorating from the point of leaving the treatment works to reaching the consumers tap. Water treatment is designed to remove or inactivate microorganisms and limit organic or inorganic nutrients, which may promote microbial growth within drinking water distribution systems (DWDS): a complex network of pipes, reservoirs and associated assets. Planktonic microorganisms may enter DWDS by surviving treatment processes or through ingress via the pipe network (normally associated with leaks, bursts or changes in demand). Once in the DWDS, microorganisms may survive as planktonic cells, but, more commonly, become incorporated into biofilms at the pipe walls. Within DWDS, the multiplication of microorganisms within the bulk water and biofilms at the pipe wall is termed regrowth. Microbial regrowth within DWDS can cause drinking water biological stability to deteriorate, potentially generating bacterial regulatory failures. Biofilms on the pipe walls of DWDS can support the multiplication of potentially pathogenic bacteria detrimental to human health [1], and reduce the biological stability (water that does not promote the growth of microorganisms during its distribution), and aesthetic quality of drinking water when biofilms are released into the water column. Additionally, microbially-mediated processes occurring within biofilms can increase the occurrence of operational issues such as biofouling and bio-corrosion.

Assimilable organic carbon (AOC) is the fraction of carbon most easily consumed by bacteria, facilitating microbial regrowth [2]. The dissolved organic carbon (DOC) fraction of TOC can be divided into two subsets; biodegradable dissolved organic carbon (BDOC) and assimilable organic carbon (AOC) (S1 Fig). AOC is made up of low molecular weight organic molecules, being generated either via lysis of bacterial cells or biological and chemical hydrolysis of organic matter. The AOC fraction of organics is small, reported to vary between 3 and 500 μg/L, representing 0.1-8.5 percent of the DOC pool [3]. AOC analysis in operational DWDS is limited due to the time and resources required to complete the original bioassay, and the variation in available AOC methods making it difficult to compare between applications.

### AOC within WTW & DWDS

AOC analysis is often used to assess the efficiency of AOC removal at the treatment works, rather than reporting concentrations within the DWDS. It is largely accepted that AOC is generally removed by the majority of conventional treatment steps, specifically biological filtration [4]. However, AOC is formed during ozonation processes as complex natural organic matter is broken down into low molecular weight by-products [5, 6]. A significant positive correlation has been found to exist between the AOC concentration and the density of heterotrophic bacteria [7, 8]. AOC has been demonstrated to limit microbial growth, and therefore constitute biostable water at concentrations spanning ≤10 – 100 μg/L [9, 10, 11, 12]. However, there is no global consensus as to the AOC concentration that constitutes biostable water, partly due to a lack of comparative AOC methods being used.

With regards to AOC values reported in distribution, Van der Kooij et al [4] and LeChevallier et al [8] demonstrated that the AOC concentration decreased with distance through the DWDS. LeChevallier et al [8] concludes that AOC was consumed by heterotrophic organisms within the DWDS. Although a correlation between AOC and HPC counts / coliforms has been identified, it is not clear what is driving such changes in AOC concentration. If SR inlet and outlet sampling is not undertaken, it is not clear if the DWDS pipes or service reservoirs (SR) are acting as sources or sinks of AOC. In addition, difficulties are presented when comparing between studies which incorporate different steps in the AOC methodology. Gibbs et al [13] for example, used a natural inoculum and acknowledged that their inoculum may not have been representative of the bacteria found within the DWDS.

#### Existing AOC methods

No direct chemical test exists to determine AOC concentrations and therefore AOC must be measured indirectly by utilising the linear relationship between the maximum growth of bacteria in a water sample and the AOC concentration. Typical steps in the AOC bioassay include: pasteurisation / filtration of the collected water sample, inoculation with a known cell density, incubation until maximum cell density is reached, enumeration and conversion of cell counts to carbon concentration. AOC analyses can be broadly split into those methods which use known bacterial strains (such as *Pseudomonas fluorescens* strain P-17 and *Spirillum* strain NOX) as an inoculum and those that incorporate a natural microbial inoculum (S1 Table). Further divisions are made based on methods that use different sample preparation steps, inoculum volumes, incubation time and temperatures, and/or enumeration methods. Generally, a yield factor is generated by monitoring the growth of the test organisms on pure solutions of acetate-carbon or oxalate-carbon. These are used as reference standards to convert cell counts to nutrient concentration. Each method utilises a different yield factor, potentially leading to large difference in the final AOC concentration and creating further difficulties in comparing AOC studies that employ different methods.

#### Known bacterial strains, P-17 and NOX

The original AOC bioassay included the use of a known bacterial strain: *Pseudomonas fluorescens* strain P-17, hereafter referred to as P-17 [7]. P-17 is used for AOC analysis as it is ubiquitous in the DWDS and able to cope with low organic carbon concentrations by metabolising a range of biodegradable compounds [14]. More recently *Spirillum* strain NOX, hereafter referred to as NOX, has been used in tandem with P-17 as it was found that NOX, unlike P-17, can degrade ozonation products [15]. Briefly, a water sample is pasteurised, inoculated with P-17, incubated at 15°C for 7 to 9 days, before the maximum growth rate is recorded using heterotrophic plate counts (HPC). Previously derived yield values of 4.1x10^6^ CFU P-17 / μg acetate carbon and 1.2x10^7^ CFU NOX / μg acetate carbon can be used to convert cell counts to AOC concentration [9]. The incubation periods required to achieve maximum bacterial growth, combined with HPC incubation (3-5 days), results in a typical sample processing period of 14 days.

Alternative methods to enumerate the bacteria have been proposed, these include: Adenosine triphosphate (ATP) luminescence [16] and bioluminescence [17, 18, 19]. LeChevallier et al [19] were able to convert ATP luminescence to AOC concentration using the linear relationship between viable cell counts and ATP luminescence units, and the yield factor for acetate carbon. The method was able to detect a greater number of bacteria than the plate count procedure and was a substantially faster enumeration method. However, it was also evident that the different test organisms did not contain the same amount of ATP per cell, consequently showing bias towards different strains and therefore not suited for the use of indigenous bacterial populations. Aggarwal et al [20] analysed the use of flow cytometry with and without SYTO9 staining to enumerate P-17 and NOX at a higher incubation temperature of 23°C. When using flow cytometric enumeration, Aggarwal et al [20] found the yield of P-17 and NOX to be lower than HPC yield factors. However, it was inconclusive if these results were due to the enumeration method, or the higher incubation temperature (23°C) used for flow cytometry. This is a knowledge gap that will be addressed in this study.

#### Natural microbial community inoculum

Modifications to the Van der Kooij et al [7] AOC bioassay have included the use of a natural microbial community inoculum in which the bacteria are indigenous to the water sample in question. Samples are subsequently enumerated using flow cytometry [21, 22] or turbidity measurements [23, 24]. The use of a natural microbial inoculum instead of P-17 and NOX has been demonstrated to result in a higher final cell count [25], suggesting that a more diverse inoculum is able to utilise a wider array for organic compounds. A higher incubation temperature (30°C) reduces the incubation time of the assay. However, a natural microbial inoculum necessitates the use of a theoretical conversion value (1 μg AOC = 1 x 10^7^ cells) [21] to convert cell concentrations to AOC concentrations, instead of relating cell concentrations to growth on acetate-carbon. Furthermore, the use a natural microbial inoculum instead of a pure culture creates additional complexity in the form of quantifying the microorganisms present and understanding the interactions between the various bacterial strains.

## Aims and objectives

This study aimed to develop a rapid, robust AOC method that can be used for routine analyses within drinking water systems. An analysis of AOC within raw, treated and distributed drinking water was conducted to quantify AOC within and between different systems. Analysis of AOC at the WTW was used to prove the method developed in this study in an environment where change in AOC is expected, and as a potential measure of treatment efficacy. The application of the AOC test to SR inlet and outlets within the DWDS aimed to understand any fluctuations in AOC within the network, and identify which areas, either pipes or SR, were acting as sources and sinks of AOC. A comparison between AOC concentration and other routine drinking water quality parameters aimed to provide a greater insight into which environmental parameters are influencing AOC concentration within the network, and assess any seasonal differences.

## Materials and methods

To develop a quicker but robust AOC method, established methods by Van der Kooij [7], LeChevallier et al [16] and Hammes and Egli [21] were assessed. Assessment of the methods included protocols that incorporated different inoculum strains, incubation temperatures and enumeration techniques. The results of this analysis were subsequently used to develop an AOC protocol which was applied to 20 Scottish Water treatment works and four of their associated DWDS. AOC concentrations were determined for raw and treated water analysed at the 20 WTW as well as water samples collected from SR inlets and outlets of the four DWDS. Each of the four DWDS were characterised by different source water qualities, treatment practices and choice of secondary disinfectant. The normality of the data was analysed using the Shapiro-Wilks test and parametric (ANOVA and Tukey) or non-parametric tests (Kruskal Wallis and two-sample Wilcoxon), as appropriate, to identify any differences in water quality parameters between experiments (p<0.05).

### Development of refined AOC protocol

An initial laboratory trial analysed differences in the yield values and AOC results obtained when using two known bacterial strains, in comparison to using a natural inoculum. Triplicate sodium acetate standards containing 0 – 1000 μg/L were used to assess the ability of both methods to enumerate different carbon concentrations. The results from this test were used to inform further trails to determine the possible options for reducing the time and resources required to complete an AOC assay by modifying the: incubation temperature, inoculation volume and enumeration technique. Although flow cytometry has been demonstrated to be a successful enumeration method when using a natural inoculum, it has not yet been used to count P-17 and NOX incubated at 15°C. In the following trial, when enumerating P-17 and NOX via flow cytometry, samples were inoculated before being incubated at three different temperatures; 15°C, 23°C and 30°C to determine the effect of incubation temperature on the maximum cell yield. To determine the effect of inoculum density in the assay the inoculum volume was increased from 500 CFU/mL of each P-17 and NOX to 10,000 CFU/mL, whilst maintaining the incubation temperature at 15°C. Cells were grown in solutions containing 100 μg acetate carbon/L and enumerated using flow cytometry.

The use of an indigenous bacterial inoculum assumes that, due to the bacterial community being diverse, the organisms would be able to grow on any AOC combination within a water sample. To investigate this, different microbial inoculums were collected from post-treated water and used to measure AOC in a number of drinking water and sodium acetate samples. The autochthonous bacterial populations were prepared as described in Hammes & Egli [21]. All trials were performed using triplicate tap water samples or sodium acetate to provide standards. Controls were included for growth, yield and negative controls for carbon contamination in all cases. The blank control consisted of inoculating a carbon free mineral salts solution (inorganics) with P-17 and NOX to check for any carbon contamination from glassware. The growth control, consisting of a water sample containing diluted mineral salts (inorganics) and diluted acetate (carbon), was used to determine if samples were limited by nutrients other than carbon. The yield control was used to check the yield of the organisms by monitoring their growth in high-performance liquid chromatography (HPLC) water containing diluted mineral salts (inorganics) and diluted acetate (carbon).

#### Glassware preparation

For the following protocols all glass was rendered organic-carbon free by following a series of washes and sterilisation. This included a wash with common detergent, rinse with distilled water, soak in 0.2 N HCl overnight, rinse with distilled water, foil cap and heat in a muffle furnace at 550°C for 6 h. Polytetrafluoroethylene (PTFE) lined silicone septa were used to cap the 45 mL borosilicate glass vials.

#### Strains, P-17 and NOX

When using the two known strains, cultures of P-17 (ATCC 49642) and NOX (ATCC 49643) were acquired from the American Type Culture Collection (ATCC). The cultures were rehydrated using Nutrient Broth (Sigma-Aldrich) (8 g/L) for P-17 (26°C for 24 hours) and Trypticase Soy Broth (Sigma-Aldrich) (30 g/L) for NOX (28°C for 72-96 hours). The cultures were stored in cryovials using 20% glycerol at -70°C. Frozen cultures were thawed and streaked out on R2A agar (Sigma-Aldrich) and incubated at room temperature for 3-5 days. The preparation of the stock cultures was performed as described in LeChevallier et al [16] and Aggarwal et al [20].

Water samples were pasteurised at 70°C for 30 minutes before being inoculated with 500 CFU/mL of pure cultures P-17 and NOX, and incubated at 15°C for 9 days, with samples being removed on days 7, 8 and 9 for enumeration. Heterotrophic plate counts (HPC) were performed by generating a dilution series before plating on nutrient or R2A agar and incubating at 25°C for 3 to 5 days. Samples enumerated using HPC counts were plated at 10^2^, 10^3^ and 10^4^ dilutions in duplicate. The average net growth of days 3, 4 and 5 was then used to generate a final AOC concentration in acetate-C equivalents using a pre-derived yield value of 4.1 x10^6^ CFU P-17/μg acetate-C and 1.2 x10^7^ CFU-NOX/μg acetate-C.

When enumerating P-17 and NOX via flow cytometry, pasteurised water samples were inoculated into separate vials with eitherP-17 or NOX, and incubated at 15°C for 7-9 days, before being enumerated using flow cytometry. Samples were enumerated using ATP as described in LeChevallier et al [16]. Measurements were made using the BacTiter-GloTM Microbial Cell Viability Assay (Promega, UK). Total ATP was established using the BacTiter-Glo reagent (G8231; Promega Corporation) and a luminometer (Tecan). ATP analysis introduces an additional conversion factor to convert ATP to cell concentrations. Relative light units (RLU) were first converted to cell concentrations using a calibration curve (LeChevallier et al. [16] proposed values of 1.85 fg/cell (P17) and 0.213 fg/cell (NOX)). The estimated cell concentrations were further converted to AOC concentrations using the proposed conversion values of Van der Kooij et al. [7].

#### Natural microbial inoculum

The natural microbial inoculum was prepared according to Hammes & Egli [21]). In short, 40 mL of water was collected from a drinking water treatment works (WTW 6 which is supplied by a reservoir source and treated using rapid gravity filtration) and filtered (0.22 μm pore, Millex-GP, Millipore - as described above) to remove particulate organic carbon. The sample was then inoculated with 100 μL of unfiltered water and incubated at 30°C for 14 days. The cells were harvested by centrifugation (10 min, 3000g (relative centrifugal force)) and re-suspended in mineral buffer. The solution was incubated for an additional seven days to ensure that all residual organic carbon had been degraded. To test the influence of different natural microbial inoculums, inoculums were collected from four different post-treatment locations. The four inoculums were subsequently used to calculate the AOC concentration within sodium acetate samples containing a known concentration of carbon.

As the inoculum used in this assay was autochonous to the water sample in question, pasteurisation was not required. Instead the water samples were filtered (0.22 μm pore, Millex-GP, Millipore - as described above) to remove bacteria and interfering particles, and re-inoculated within 10 μL of unfiltered water. Samples were enumerated at 30°C until stationary phase is reached. A 1 mL volume of samples are stained with 10 μg/mL of SYBR Green (Life Sciences) and enumerated with flow cytometry. Where necessary, samples were diluted after staining in filtered (0.22 μm) mineral water, so that counts measured were always less than 3 x 10^5^ cells/mL. To convert the cell counts to AOC concentrations, a theoretical conversion value of 1x10^7^ cells was used [21]:

$$AOC\;(\mu g\;C/L)=\frac{\left(net\;grown\;cells)/L\right)}{1\;x\;10^{7}\;cells/\left(\mu g/C\right)}$$

### Application of AOC method

In order to a) demonstrate the ability of the AOC test to quantify a wide range of AOC concentrations b) potential measure of treatment efficacy, the developed AOC method was applied to raw (pre-treatment) and treated (post-treatment) drinking water samples collected from 20 WTW on a weekly basis for two months. WTW were selected with contrasting source waters, treatment processes and disinfectant types to reflect a large array of AOC concentrations.

**Fig 1:**
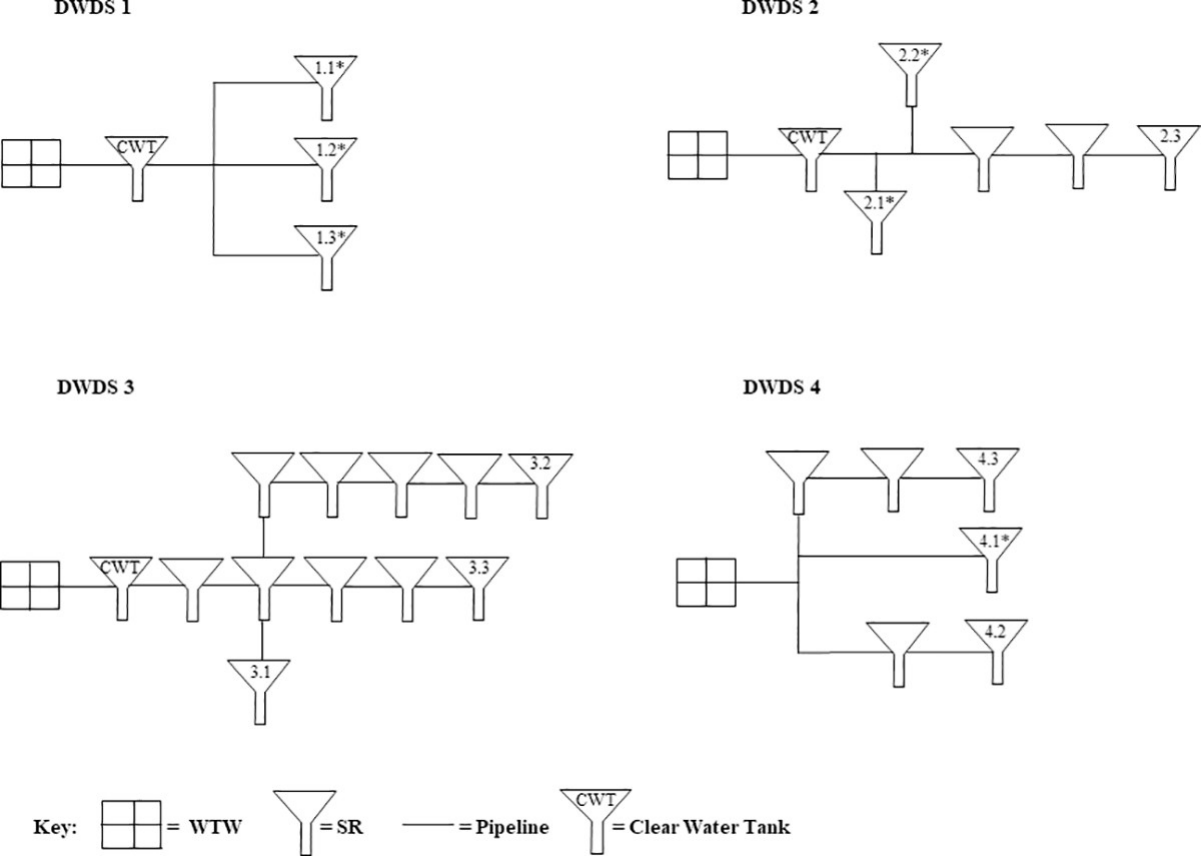
Schematic of the arrangement of service reservoirs (SR) within the four DWDS selected for further sampling (not to scale). The SRs sampled for AOC are numbered from one to three in each distribution system, as indicated by the second number in each case with the first number denoting the DWDS to which the SR belongs. Unlabelled SRs are not sampled as part of this study and are only drawn to show the pathway of the water. SRs labelled with a * are subject to the pipe only effect, as water has not previously passed through a SR. Samples analysed included: WTW (raw): AOC, TCC & ICC; WTW (post-treatment): AOC, TCC, ICC & total chlorine; SR inlets: AOC; SR outlets: AOC, TCC, ICC & total chlorine.

As highlighted in Table 1, from the twenty WTW, four were selected for more in-depth AOC sampling at the treatment works and from three SR with increasing distance through the DWDS network (henceforth referred to as DWDS 1-4) (Fig 1). The four DWDS were selected for AOC sampling based upon their different characteristics, specifically: different source waters, treatment processes, disinfection residuals and size of distribution zones. The AOC concentration at each of the four DWDS was determined bi-weekly for 12 months to investigate spatial and temporal trends in AOC.

**Table 1:** Twenty WTW selected for AOC sampling within raw (pre-treatment) and final (post-treatment) water [10]. The final column represents the 4 DWDS selected for further AOC sampling at service reservoirs inlet and outlets.

Source Water	Treatment Type	WTW Identification Number	Disinfectant Type	DWDS Identification Number*
Groundwater	Conditioning only	1, 2, 3	Cl	-
	Membrane	4	NH_2_Cl	DWDS 1
Reservoir	RGF	5, 6, 7, 8,9	Cl	DWDS 2
	DAF	10		-
	GAC	11		-
	Membrane	12		-
		13	NH_2_Cl	-
River	Clarification	14	NH_2_Cl	-
	RGF	15, 16, 17	NH_2_Cl	DWDS 3
	DAF	18	Cl	-
	Membrane	19		-
		20	NH_2_Cl	DWDS 4

*DWDS 1-4 were selected for investigation as they contain different AOC concentrations in post treated water.

WTW = water treatment works; DWDS = drinking water distribution system. Table includes water source, treatment type (RGF: rapid gravity filter, DAF: dissolved air flotation, GAC: granular activated carbon, or membrane), choice of disinfectant (Cl: chlorine, NH2Cl: monochloramine) and number of WTW with each treatment type.

DWDS pipes and SR are characterised by different volume to surface areas. To ensure that pipe and SR effects on AOC could be clearly differentiated SR inlet and outlet points were sampled. The 12 SR sampled for AOC (labelled in Fig 1) were located 3.35 to 53.26 km into the network. The various pathways that the water travels to reach each of the 12 SR are shown in Fig 1. In some cases water passes through a series of pipes and SR to reach the sampled SR, and in some cases only pipes.

Water samples for AOC testing (n = 3) were collected using AOC-free glassware from raw and post-treated water at the works, and from each of the three SR inlets and outlets as indicated in Fig 1. Additional water samples were collected for total chlorine, total cell counts (TCC) and intact cell counts (ICC) from raw and post-treated water at the WTW, and from each of the three SR outlets. Additional water samples were measured as described in S2 Table. It should be noted that no total chlorine, TCC or ICC data was collected for SR inlets as the main focus of this study was to capture the effect of large volumes and residence times of the SR by sampling at the outlet. Total and intact cell count data was not collected for DWDS 1. All water samples were analysed within 24 hours.

## Results

### Evaluation AOC assays

**Fig 2:**
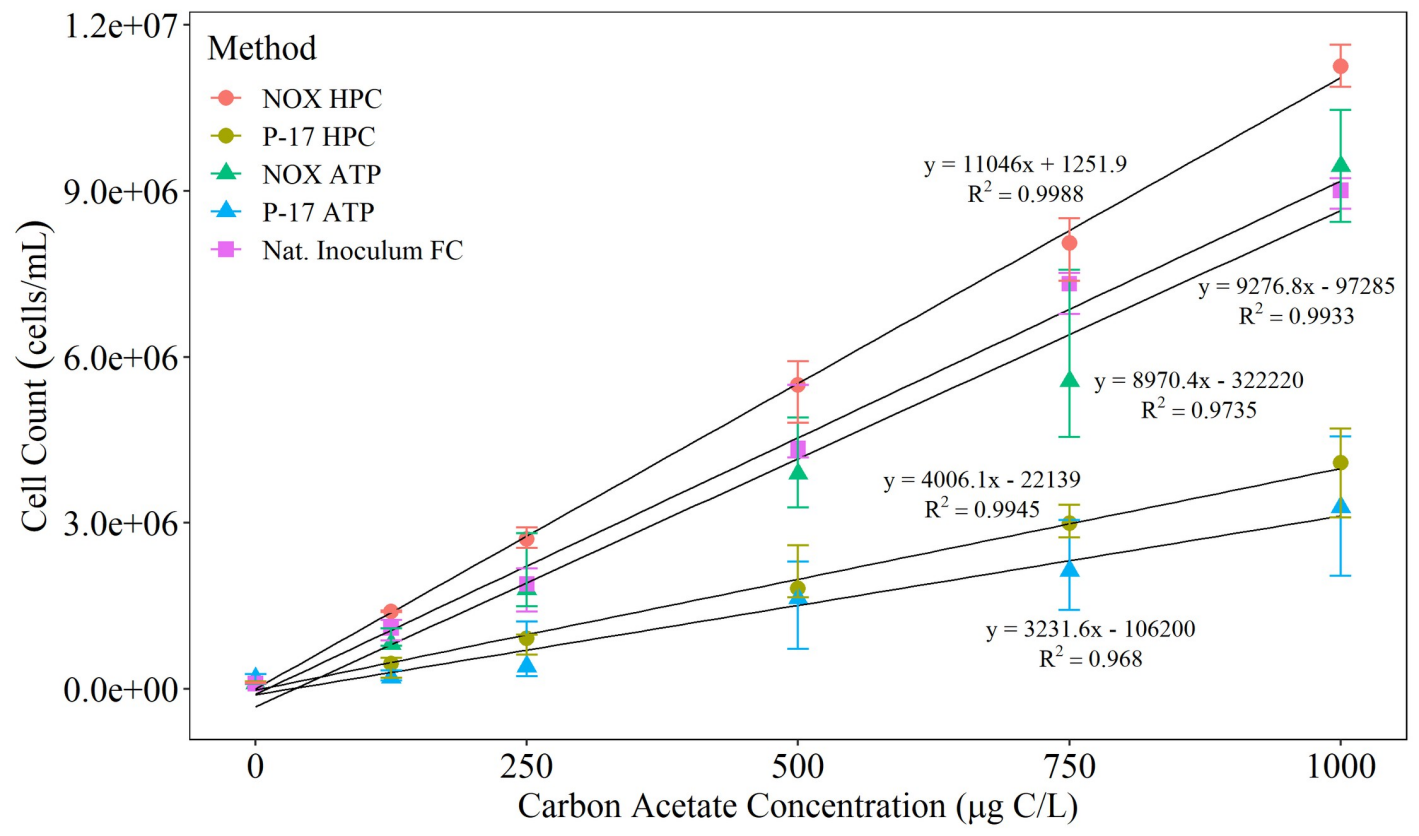
Comparison of cell counts enumerated using bacterial strains NOX (NOX HPC) and P-17 (P-17 HPC) with heterotrophic plate counts (HPC) or adenosine triphosphate (ATP), or using a natural microbial inoculum with flow cytometry (Nat Inoculum FC). HPCs are recorded as CFU / mL and total cell counts (TCC) are recorded as cells / mL. ATP luminescence units converted to cells / mL using an ATP calibration curve. The average (n = 3) cell counts (± standard deviation) at each acetate carbon concentration are plotted. The flow cytometry count refers to the total cell count.

Fig 2 compares the yield curves produced by using bacterial strains NOX and P-17 enumerated with plate counts (Van der Kooij [7] protocol) and ATP (Lechevallier et al [16] protocol), compared to a natural microbial inoculum enumerated using flow cytometry (Hammes & Egli [21] protocol). This trial was conducted to determine how two existing AOC methods perform and enable a comparison between them. Flow cytometry and HPC enumeration methods showed good reproducibility between triplicate measurements. The ATP method generated a higher degree of standard error between measurements than flow cytometry and HPC, and consistently underestimated cell counts. Comparison between the datasets for the Van der Kooij [7] protocol and Lechevallier et al [16] protocol resulted in a p-value = 0.002, indicating that the results of the methods significantly differed. The ATP enumeration method was therefore not used in further AOC trials. Using a natural microbial inoculum resulted in a higher final cell count per mL compared to using P-17 counts in the Van der Kooij et al [7] protocol. Additionally, the growth rate of the natural microbial inoculum was very different from that of P-17. Ultimately the time consuming nature of enumerating known strains with HPC demonstrated the need for an improved AOC method.

### Development of adapted AOC method

**Fig 3:**
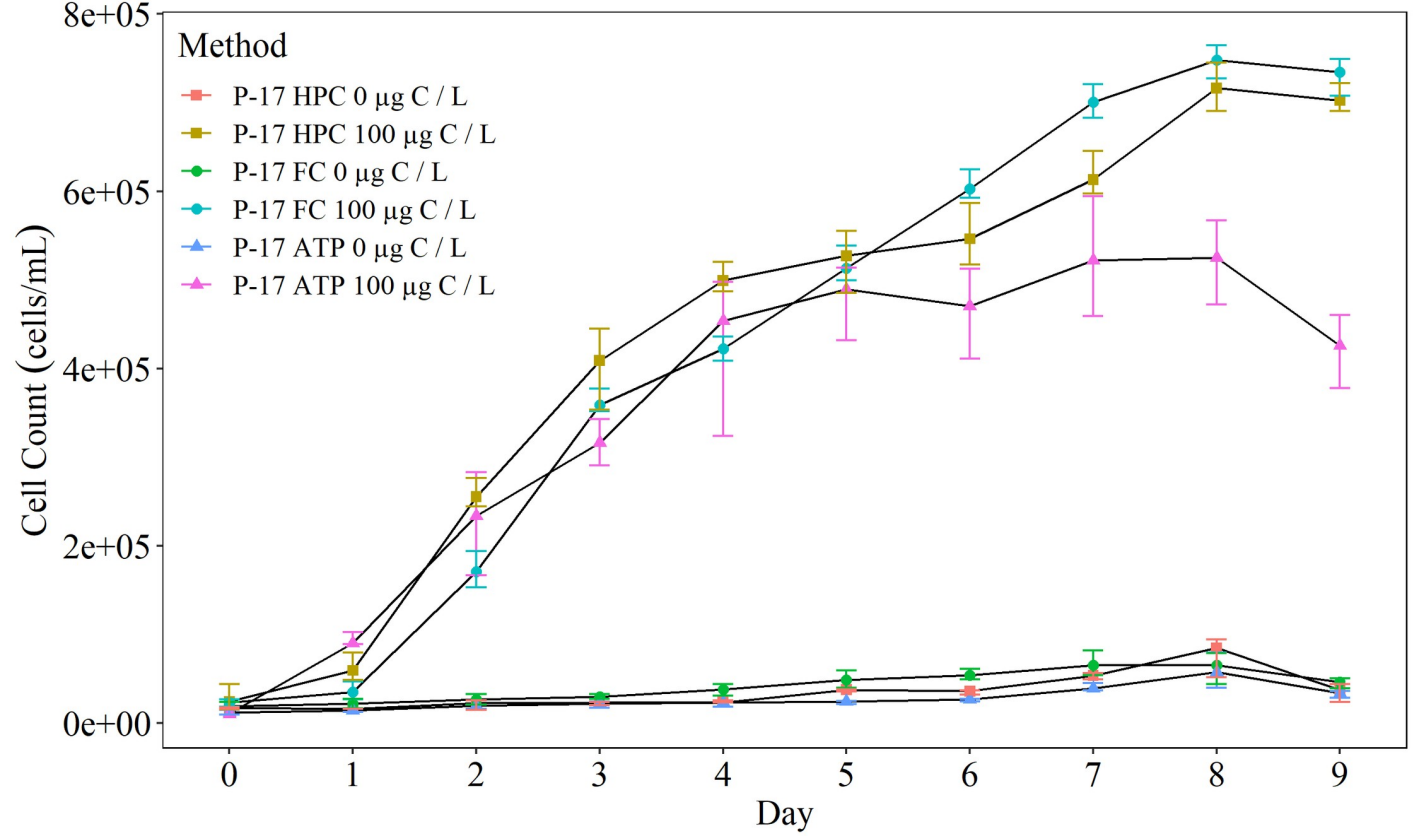
Comparison of P-17 cell concentrations when enumerated using heterotrophic plate counts (HPC), flow cytometry (FC) or adenosine triphosphate (ATP) over a nine day period. Cells are grown on in solutions containing 0 μg acetate carbon / L (control), and 100 μg acetate carbon / L. HPCs are recorded as CFU / mL and total cell counts (TCC) are recorded as cells / mL. ATP luminescence units converted to cells / mL using an ATP calibration curve. The average (n = 3) cell counts (± one standard deviation) are plotted.

To assess the repeatability and accuracy of enumeration methods, the ability of HPC, flow cytometry and ATP to enumerate P-17 was first assessed. As demonstrated in Fig 3, flow cytometry results showed equal or smaller variance between triplicate measurements, with greater consistency than HPC and ATP enumeration. The same experiment was performed using NOX (not plotted).

**Fig 4:**
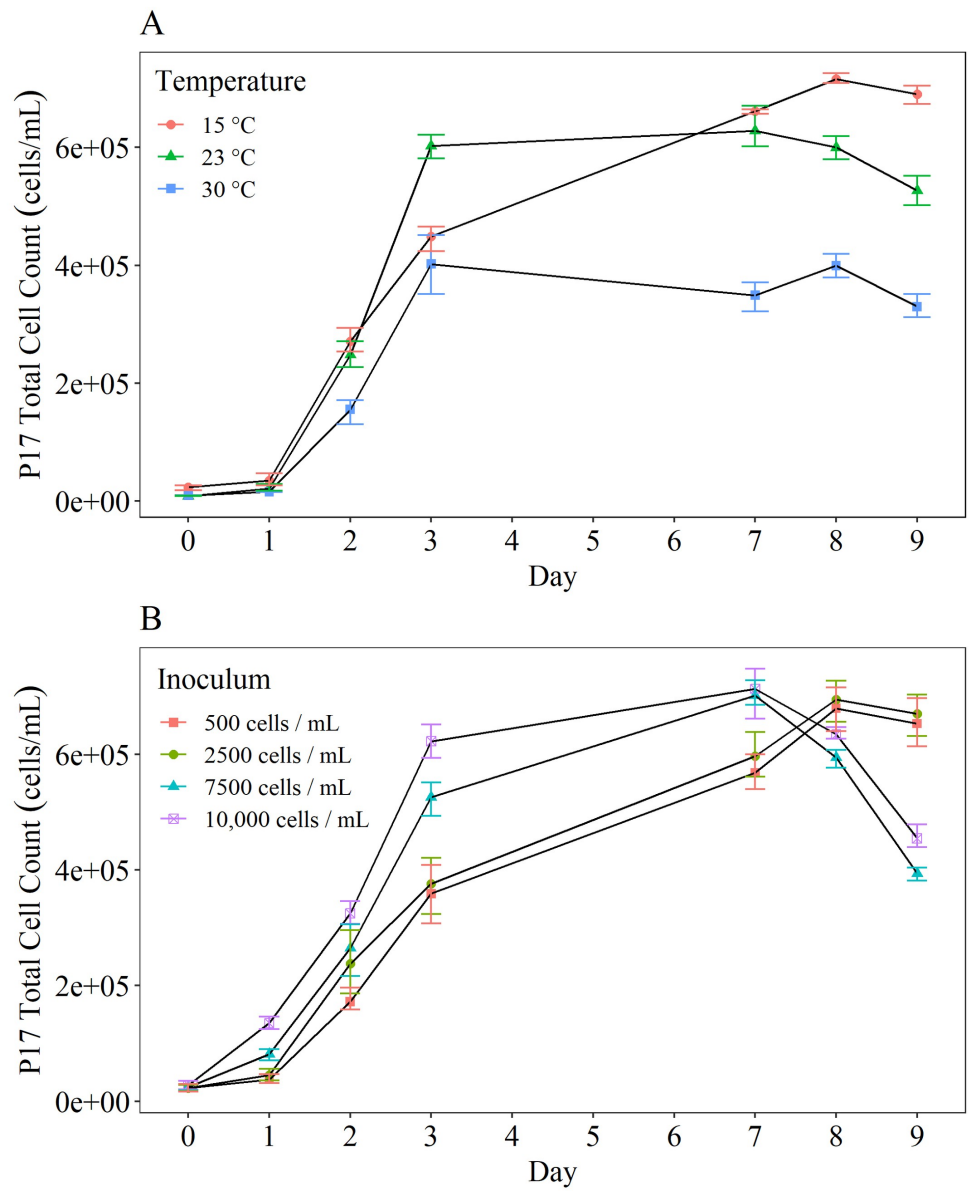
**Comparison of P-17 cell growth over a 9 day period when incubated at (A) different temperatures and (B) with different inoculum densities.** All samples were enumerated using flow cytometry and recorded as cells/mL. Cells were grown in solutions containing 100 μg acetate carbon/L. (A) Inoculum density was 500 cells/mL, temperature as indicated by the key. (B) Temperature was 15°C, inoculum density as indicated by the key. The average (n = 3) cell counts (± standard deviation) are plotted.

Testing different incubation temperatures for P-17 showed that the stationary phase of growth (rate of bacterial cell growth is equal to the rate of bacterial cell death) was reached more quickly at 30°C than 15°C, but the peak number of cells (N-max) was lower at 30°C (Fig 4A). The same experiment was performed using NOX (not plotted). The maximum number of cells was reached when the sample was incubated at 15°C, suggesting that this is the optimal temperature for P-17 growth. Ultimately, this data highlights that temperature variations generate different yield factors. Maintaining the P-17 cultivating at 15°C but increasing the inoculum density reduced the time taken to reach the stationary phase of growth but did not affect the maximum cell count (Fig 4B). Using a natural inoculum of 10,000 was found to be statistically different from using an inoculum volume of 500 (p = 0.0001) and 2,500 (p = 0.001). Therefore an inoculum volume of 10,000 CFU/mL will be used for subsequent tests.

**Fig 5:**
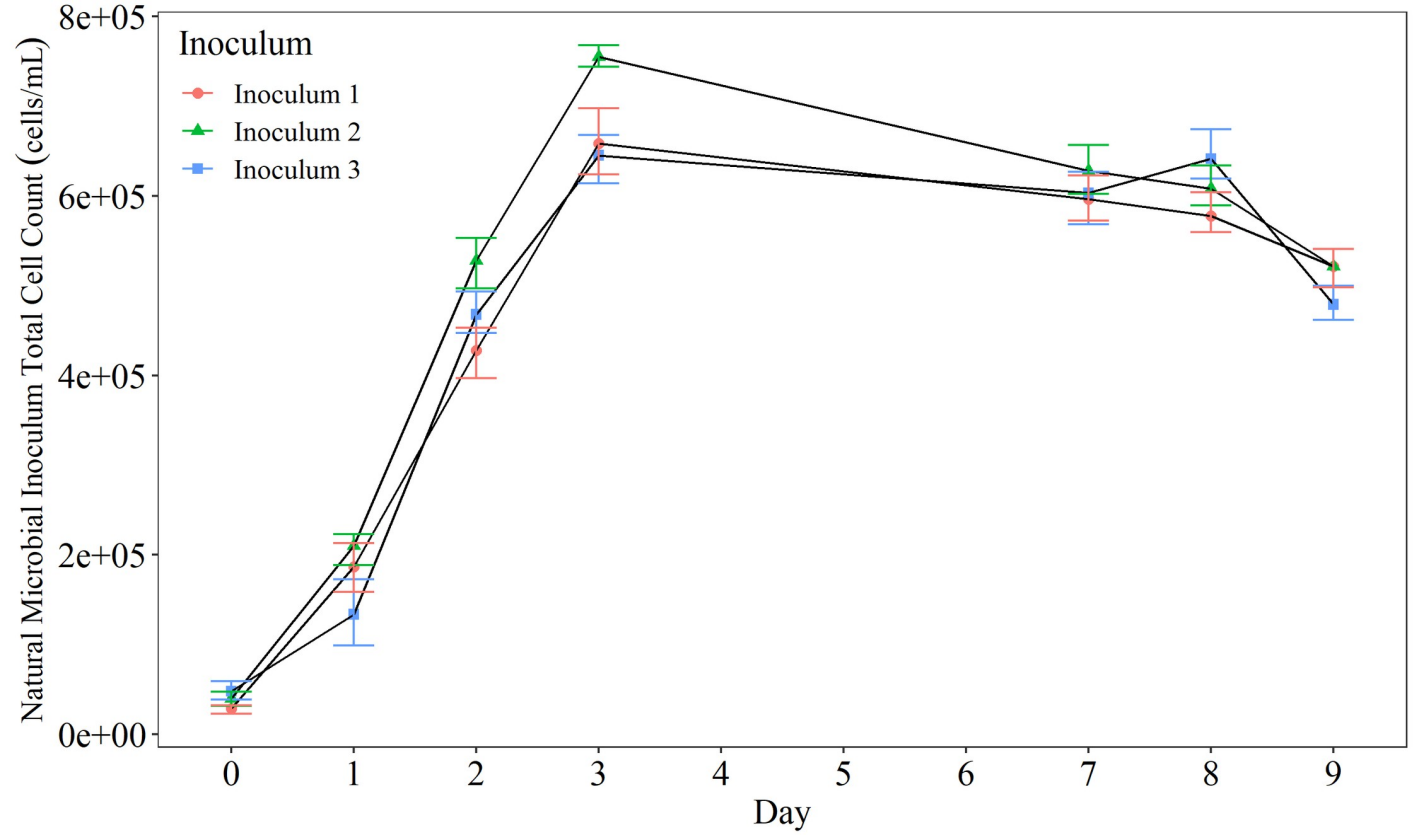
The effect of sample location on natural inoculum growth rate. The three inoculums were collected from three separate treated (post-treatment) water locations and prepared according to Hammes & Egli [21]. Inoculums were added to separate solutions containing 100 μg acetate carbon/L and incubated at 30°C for 9 days. Cell enumeration was via flow cytometry. The average (n = 3) cell counts (± standard deviation) are plotted.

A final preliminary test was conducted to assess the reproducibility of using a natural inoculum in the AOC test (Fig 5). As alternate strains of bacteria have different growth rates, it is important to assess the consistency of growth rates resulting from different inoculums. Natural inoculums were obtained from three separate drinking water sources characterised by different conventional treatment processes to obtain a diverse natural inoculum (post treated / filtered water from WTW 6, 13 and 14).

**Fig 6:**
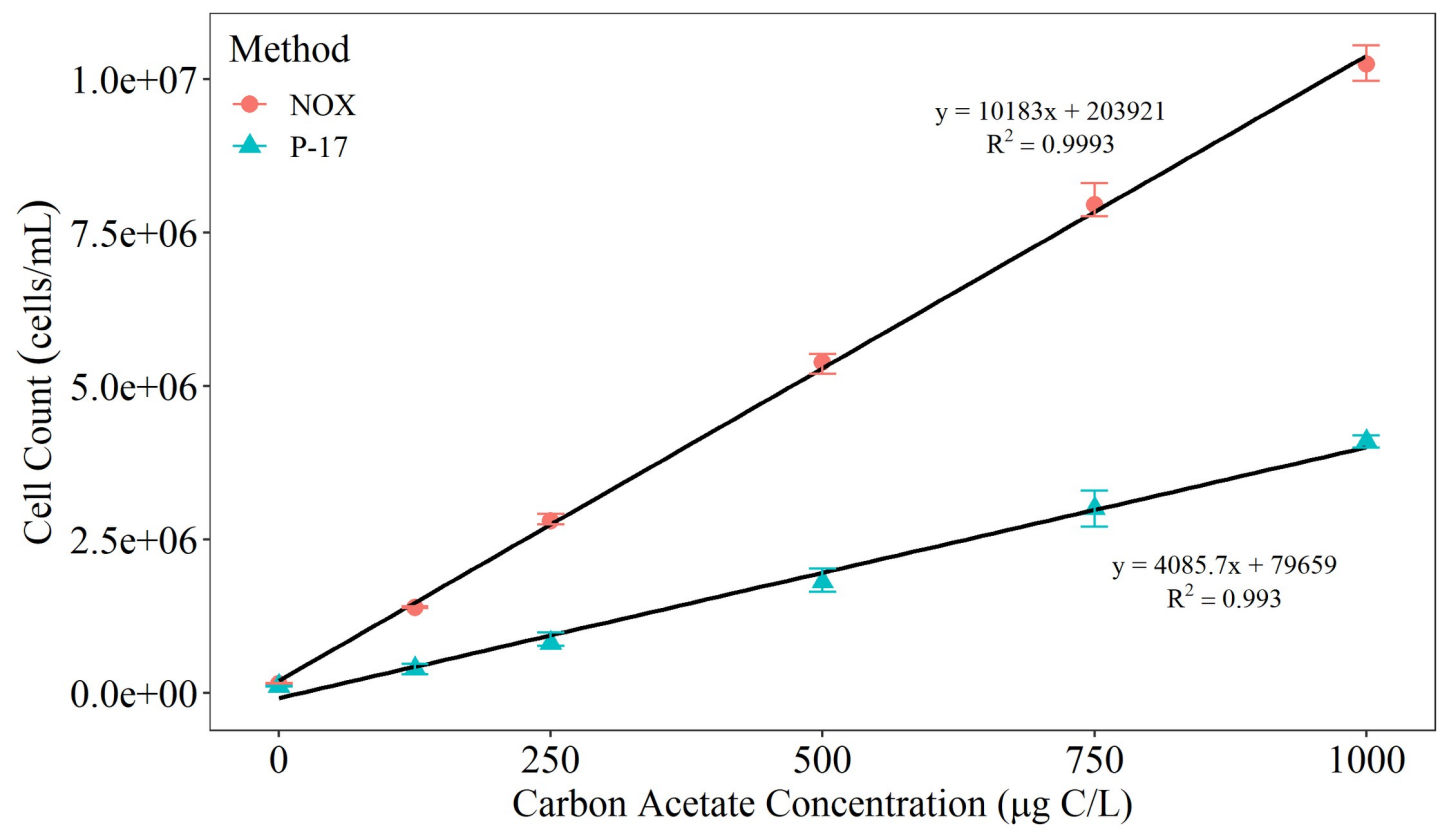
Yield factors produced when using NOX and P-17 grown in 0 – 1000 μg/L sodium acetate solutions. Samples were inoculated with 10,000 cells ml^-1^, incubated at 15°C until maximum cell growth was achieved and enumerated using flow cytometry. Total cell counts are presented as averages ± standard deviation. The average (n = 3) cell counts (± standard deviation) are plotted.

The different natural microbial inoculums resulted in a statistically different (p = 0.0015) growth rate for Inoculum 2, most likely due to differences in their microbial community composition. Inoculum 1 and 2 were not found to be statistically different with p values = 0.3184 and 0.2891 respectively. As a natural microbial inoculum needed to be changed at least every month, this would make standardising the method difficult. Consequently it was desirable to create an adapted method that incorporates the use of bacterial strains P-17 and NOX to increase the reproducibility of the test, coupled together with flow cytometric enumeration to increase the speed. The yield factors generated by monitoring the growth of the test organisms on pure solutions of acetate-carbon (P-17) or oxalate-carbon (NOX) are exhibited in Fig 6. These were subsequently used as reference standards to convert cell counts to AOC.

The developed AOC methodology combined the known strain inoculum approach, with a larger inoculum volume and flow cytometric enumeration to increase the speed and reproducibility of AOC concentration. In summary, 40 ml water samples were inoculated with 10,000 CFU/mL of either P-17 or NOX into separate vials and incubated at 15°C. Samples were enumerated using flow cytometry on days 6, 7 and 8. The averaged cell counts on day 6, 7 and 8 were converted to AOC values using pre-derived yield value of 4.1 x106 CFU P-17/μg acetate-C and 1.2 x107 CFU-NOX/μg acetate-C (Van der Kooij et al. [7]). By using a higher inoculation volume and flow cytometry the total AOC test time is reduced from 14 to 8 days.

### Application of the AOC assay

#### AOC at the treatment works: raw vs. treated water

**Fig 7:**
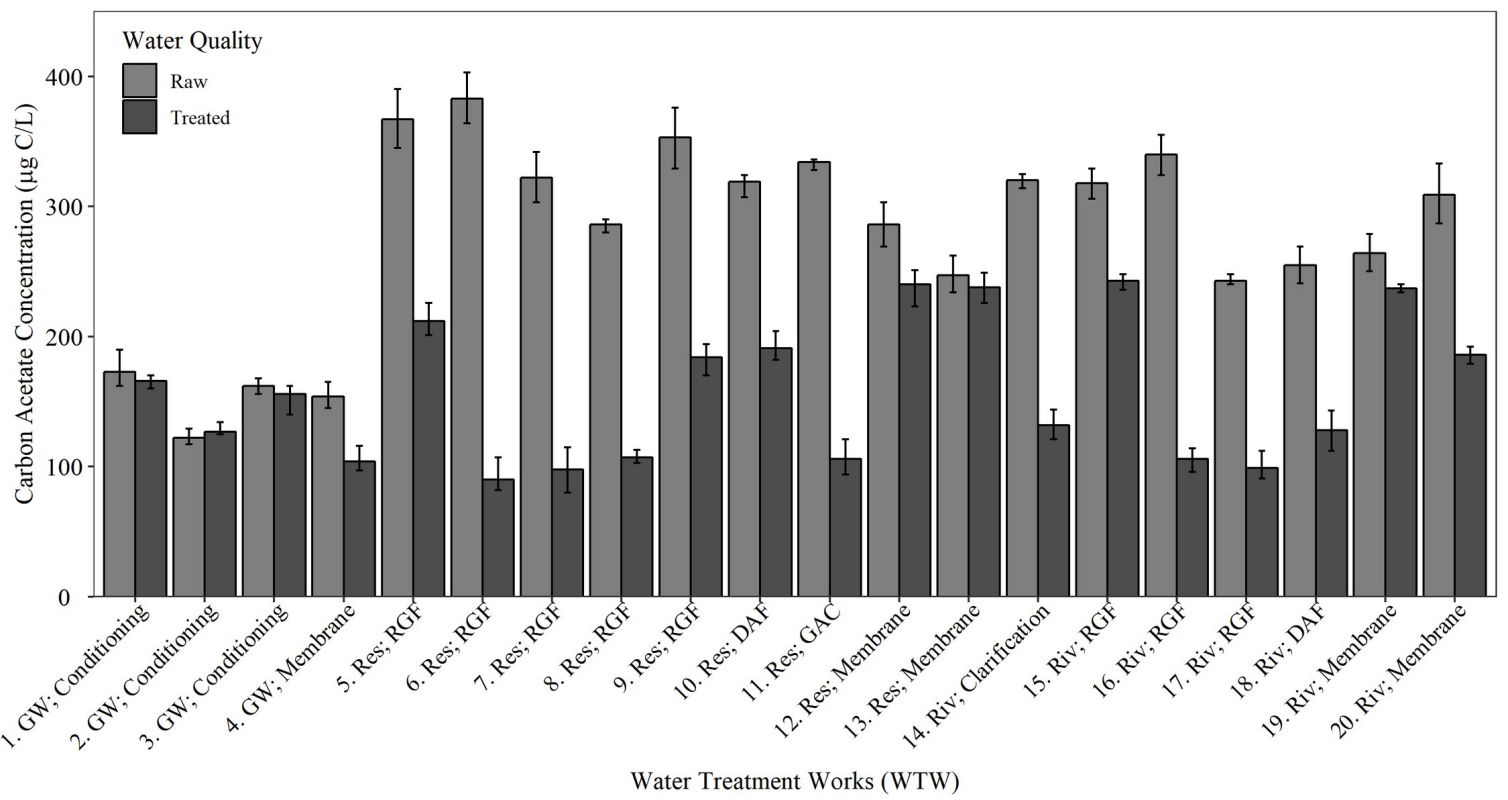
AOC concentration in raw (light bar) and treated (dark bar) water at 20 WTW (Table 1 for details of source water, treatment type and disinfectant) [26]. Data collected weekly over a two month period, average is presented (n = 24) ± standard deviation. Figure axis include the identification (ID) number of each WTW (1-20), the water source (GW = groundwater, Res = reservoir, Riv = river) and treatment type (RGF = rapid gravity filter, DAF = dissolved air flotation, GAC = granular activated carbon).

To assess the ability of the AOC method developed in this study to capture a wide range of AOC concentrations, and quantify treatment efficacy, the developed AOC method was used to quantify AOC removal at 20 WTW (Table 1), by measuring AOC in raw (pre-treated) and final (post-treatment) water. Application of method yielded informative, repeatable results, confirming the AOC test was able to capture AOC reduction at the WTW. The AOC concentration decreased post-treatment at every WTW during the treatment process with an average of 45% removal of AOC across the 20 WTW sampled (Fig 7). The AOC concentrations in post-treated water were found to be more dependent on incoming water quality than the specific treatment process employed. It was found that WTW supplied from groundwater sources (WTW 1-4) contained, on average, 50% less organic carbon in both raw and post treated water than their surface water counterparts (WTW 5-20). The AOC concentration within raw surface water (234-403 μg/L) was much higher than raw groundwater (117-190 μg/L). Among the water treatment works supplied by groundwater water, the AOC concentration in post-treated water ranged from 61-96 μg/L, with all of the groundwater WTW having an AOC concentration in treated water below the threshold for biostable water (<100 μg/L).

#### AOC concentrations within DWDS: AOC concentration & hydraulic retention time

**Fig 8:**
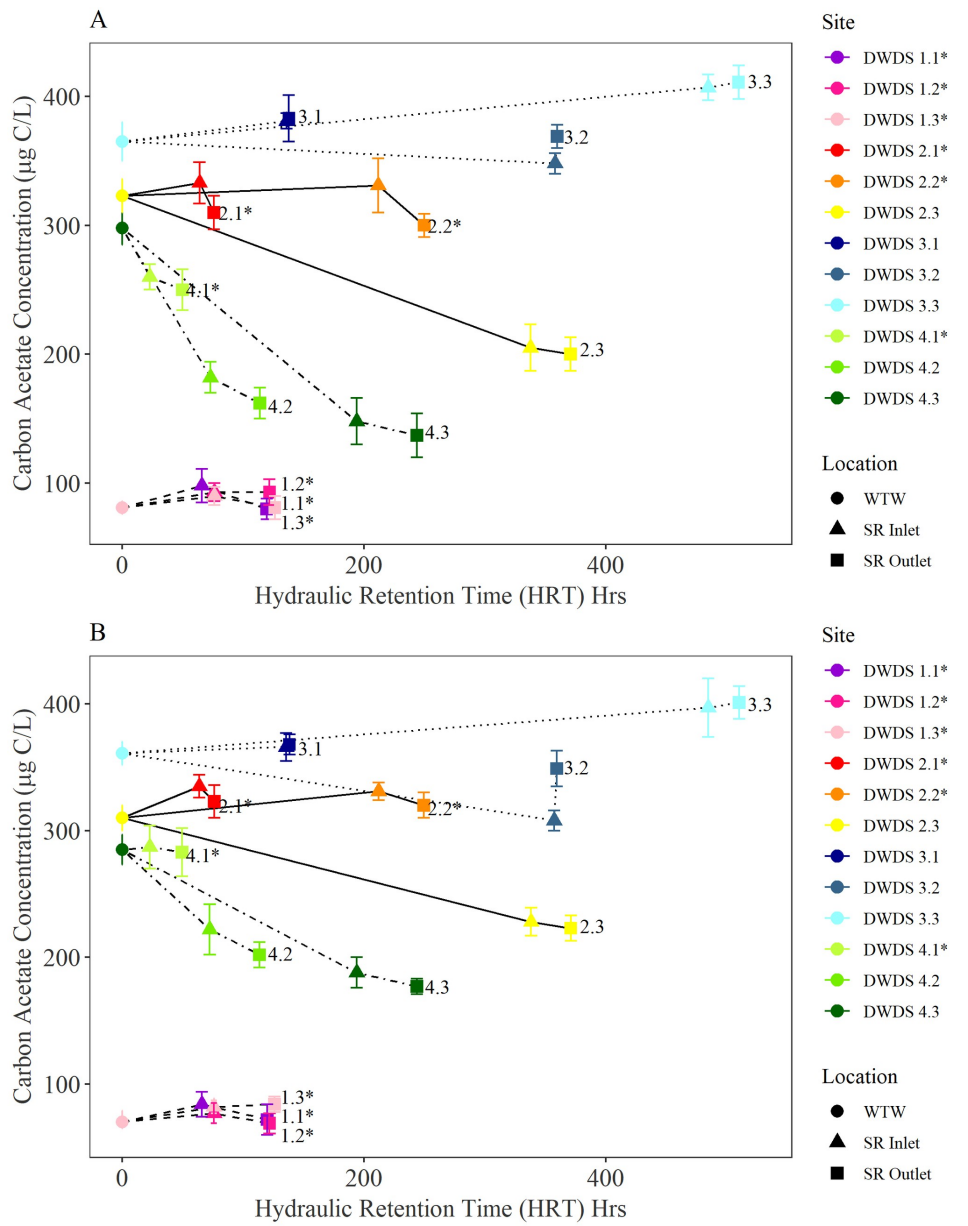
**Variation in the AOC concentration along 4 DWDS during A) summer and B) winter (** **Table 1** **for DWDS details). AOC plotted with respect to the time that water has spent in the network, i.e. hydraulic retention time (HRT).** Hydraulic residence time is calculated from the pipe and/or SR volume divided by the flow rate (l/s). Locations of samples are indicated by the key and follow the sequence post-treatment (WTW outlet) and through 3 service reservoirs (SR), inlet and outlet. Data is the annual average ± standard deviation. A network schematic of the SRs within each system is provided in Fig 1. SR marked with a * are pipe only systems (water does not pass through an un-sampled SR).

To assess AOC variability within the network, and identify which areas, either pipes or SR, were acting as sources and sinks of AOC, the AOC test was applied to drinking water samples collected from SR inlet and outlets within four DWDS. The DWDS SR connectivity, as set out in Fig 1, is captured by the lines linking points in Fig 8A & Fig 8B. AOC is plotted against hydraulic residence time rather than distance in the network to demonstrate that AOC variability is a kinetic process. Results are divided into AOC concentrations for summer (Fig 8A) and winter (Fig 8B). AOC concentration was found to be dependent on source water quality, seasonality (temperature), hydraulic residence time and pipe/SR configurations. The seasonal post-treated and SR water temperature of the four DWDS sampled are presented in S3 Table. DWDS 1, supplied by groundwater, exhibited the smallest change in temperature during the 12 month study period (range: 5.7 – 11.8°C), in comparison to DWDS 2 (4-16°C), DWDS 3 (3.8-16.5°C), and DWDS 4 (4.2-16.5°C), which are all supplied by surface water.

When comparing the AOC concentration in post-treated water to SR outlet water, AOC was found to decline within the majority of DWDS (7 out of 12 in both summer and winter). The mean AOC concentration within DWDS 2 and 4 was found to decline significantly with increasing hydraulic retention time (34.97% in DWDS 4, 34.40% in DWDS 2). Notable exceptions include DWDS 3.1 and 3.3, where AOC is found to increase. The AOC concentration within DWDS 1, supplied by groundwater, was low compared to other systems and remained relatively constant in post-treated water and through the network (60-90 μg/L during summer and 72-111 μg/L during winter).

As identified in Fig 1, the 12 DWDS consist of 6 DWDS which are pipe only (water does not pass through a SR before reaching the final sampled SR). Interestingly, when comparing AOC concentration in post-treated water to SR inlet values, the AOC concentration was found to increase within all of the pipe only sections during winter and 83% of pipe only sections during summer. In contrast, the AOC concentration was found to decrease within the majority of SRs sampled (75% in summer and 67% in winter). In DWDS 2, the AOC concentration was found to increase from the WTW to the SR inlet within DWDS 2.1 and DWDS 2.2; the two DWDS which are dominated by pipe only effects. In contrast, the AOC concentration declined from the WTW to the SR inlet of SR 2.3. In DWDS 2.3 water passes through multiple SR before reaching the inlet, suggesting a decline in AOC concentration during SR residence time. This trend was also identified in DWDS 3 as AOC was found to decline from leaving the WTW to reaching SR 3.2. During this time the water passes through 6 SR before reaching the final sampled SR.

During winter, the AOC concentration was found to decline from leaving the WTW to reaching the inlets of both SR 4.2 and SR 4.3. However, in SR 4.1 AOC was found to increase before reaching the SR. Water reaching SR 4.2 does not pass through any other SRs before reaching SR 4.2 and is therefore dominated by pipe only effects.

#### AOC, cell counts and disinfection

**Fig 9:**
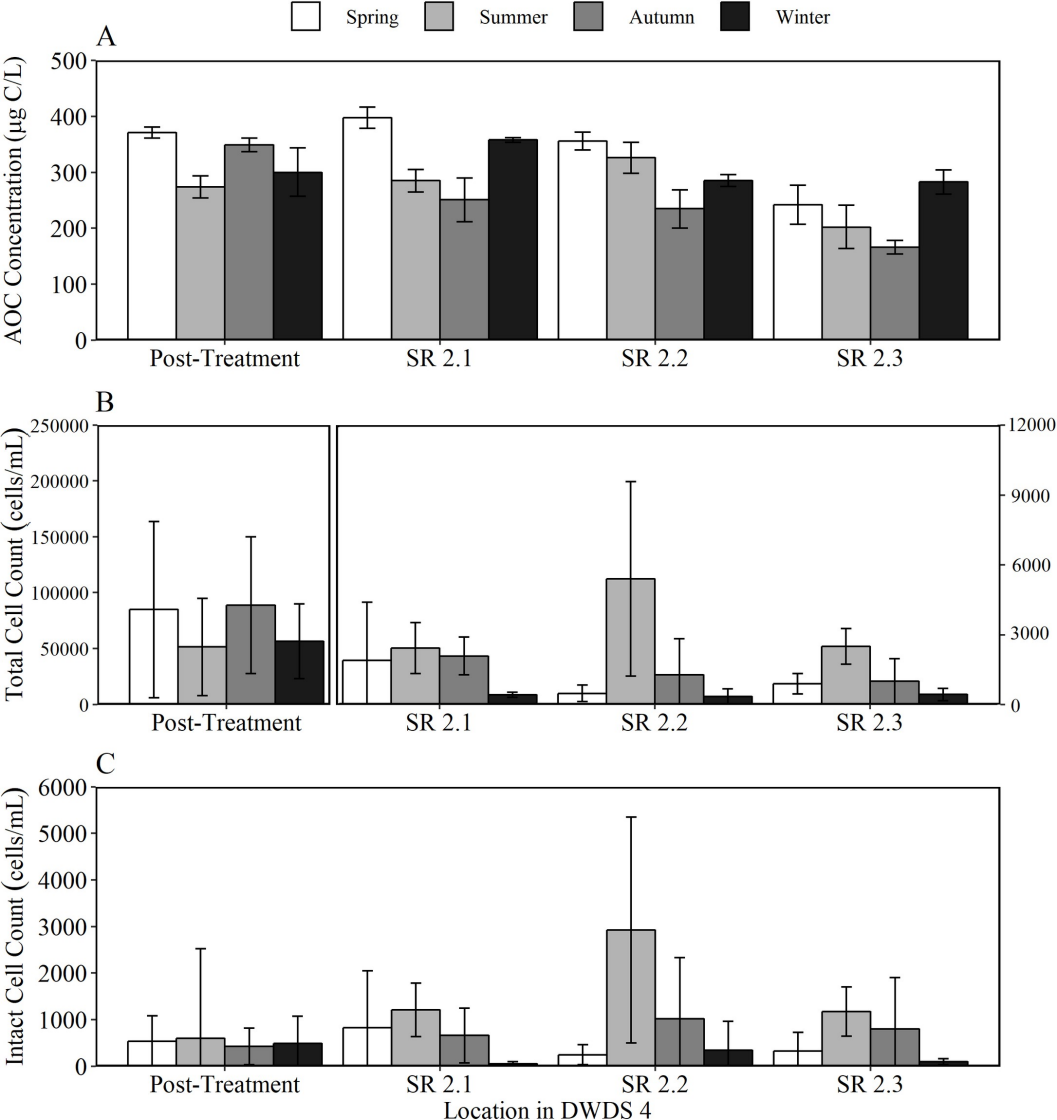
Seasonal water quality data for DWDS 2. Variation in the mean a) AOC concentration b) total cell counts (TCC) and c) intact cell counts (ICC) in post-treated water and three service reservoirs (SR) within the DWDS. Nb different y-axis scale for each parameter, and different y axis scales used in Fig 9, Fig 10 and Fig 11. Panel B of Fig 9 has two y-axis so that both the TCC within post-treated water, and each of the SR, are visible. Data was split into spring, summer, autumn and winter seasons based on monthly averages in temperature.

**Fig 10:**
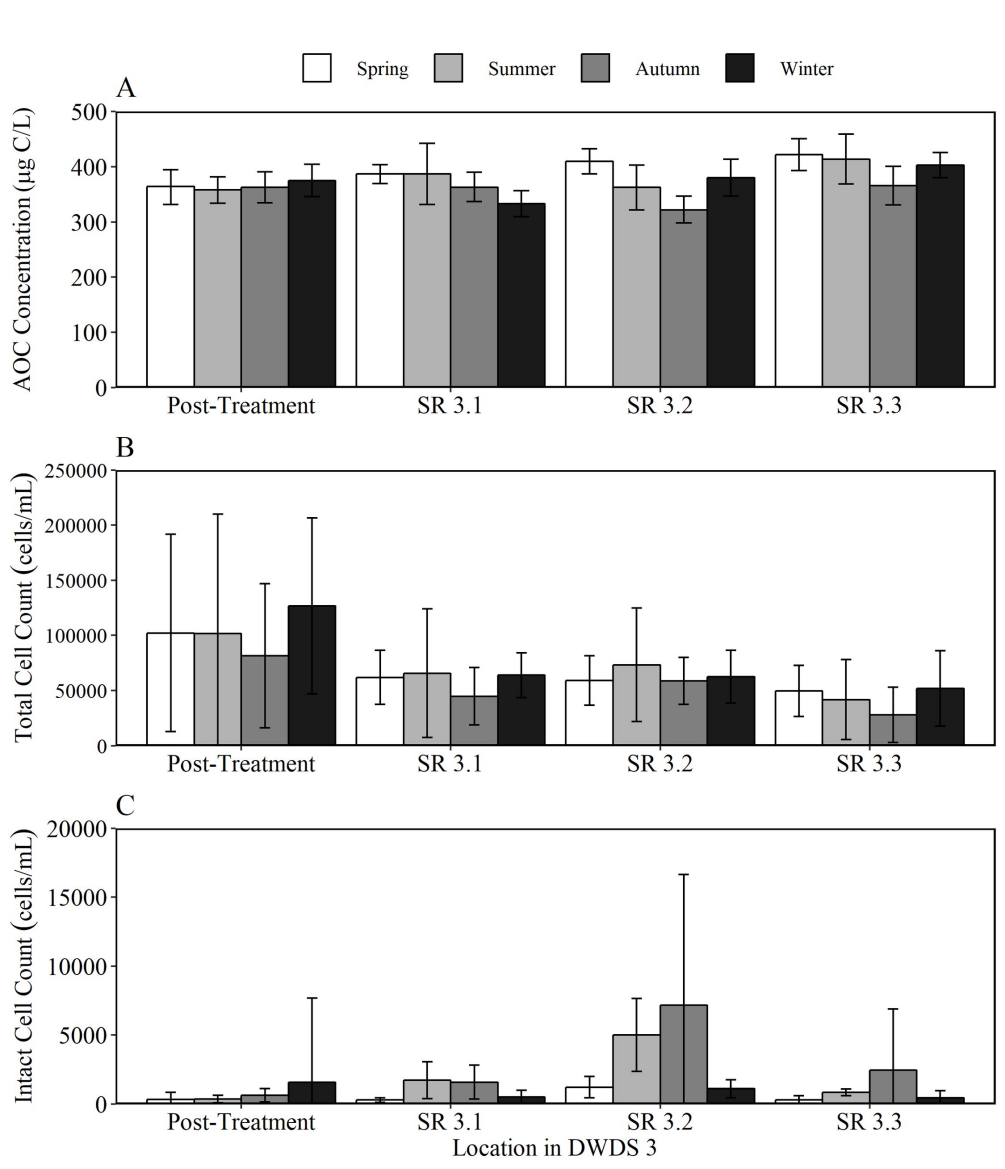
Seasonal water quality data for DWDS 3. Variation in the mean a) AOC concentration b) total cell counts (TCC) and c) intact cell counts (ICC) in post-treated water and three service reservoirs within the DWDS. Nb different y-axis scale for each parameter, and different y axis scales used in Fig 9, Fig 10 and Fig 11. Data was split into spring, summer, autumn and winter seasons based on monthly averages in temperature.

**Fig 11:**
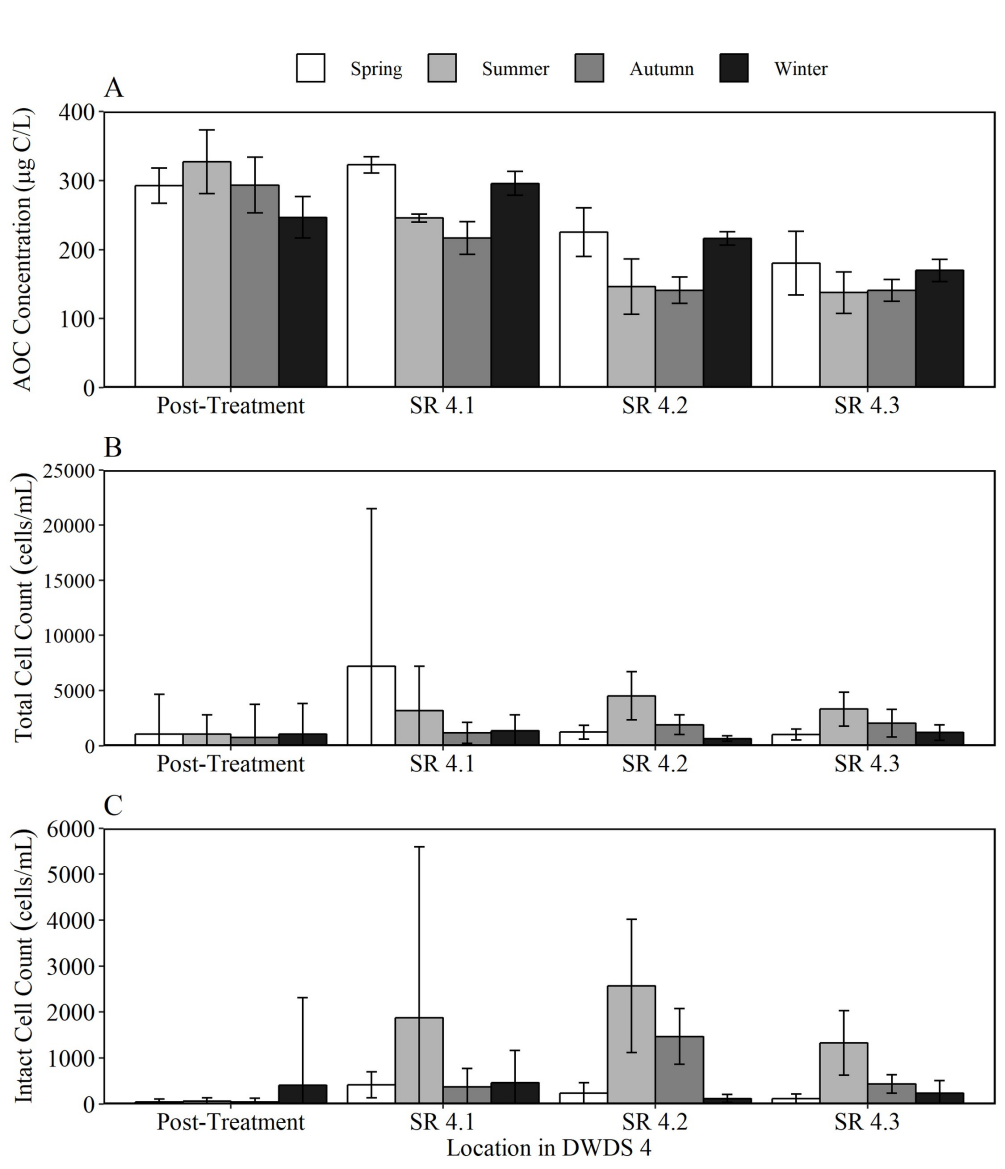
Seasonal water quality data for DWDS 4. Variation in the mean a) AOC concentration b) total cell counts (TCC) and c) intact cell counts (ICC) in post-treated water and three service reservoirs within the DWDS. Nb different y-axis scale for each parameter, and different y axis scales used in Fig 9, Fig 10 and Fig 11. Data was split into spring, summer, autumn and winter seasons based on monthly averages in temperature.

To provide an insight into the relationship between AOC concentration, cell counts and disinfection residual within the bulk water, and assess the degree of biological stability, AOC results were compared with total cell count (TCC), intact cell count (ICC) and chlorine concentration data for DWDS 2 (Fig 9), DWDS 3 (Fig 10) and DWDS 4 (Fig 11). DWDS 1 was excluded from this analysis, as flow cytometry data was unavailable. Three SR in each DWDS were analysed, as outlined previously (Fig 1). The relationship between AOC and microbial load was assessed over 12 months to firstly determine the impact of AOC concentration on cell growth, and secondly, to identify any seasonal trends in the AOC concentration and other water quality data.

The AOC concentration within DWDS 2 and DWDS 4 exhibited a seasonal trend, with AOC concentration being elevated in spring, before declining through summer and autumn (Figs 9 & 11). AOC concentrations also decreased from post treatment to final SR sampled in this study. In DWDS 2, TCC’s were high in post-treated water before declining and continuing to stay low throughout the DWDS (TCC’S 98% lower in DWDS 4.3 than post-treated water). Summer regrowth was evident within DWDS 2 with a sharp increase in ICC to 7240 cells/mL within SR 2.1. This correlated with a decline in the AOC concentration. DWDS 2, a chlorinated system, exhibited a 55.32% decline in total chlorine concentration from post-treatment to SR 3.3.

Despite very low percentages of intact cells being found in the treated waters of all three DWDS, these percentages were seen to increase with hydraulic retention time in the distribution systems. The most significant increase in the percentage of intact cells was found within chlorinated DWDS 3 with the percentage of intact cells increasing from 2.40% to over 50%. A significant decline in AOC concentration was found in SR 3.2, which correlates with an increase in ICC, especially during autumn. As with DWDS 2, some regrowth was evident within DWDS 3, especially in SR 3.2. However, the percentage of intact cells remained relatively low in DWDS 3 (1.89 to 4.12%), despite the TCC being, on average, 10 fold greater than TCC in DWDS 2 and 4. The chloraminated DWDS 3 exhibited a decline in chlorine concentration of 30.56%.

The AOC concentration within DWDS 4 was lower than DWDS 2 and 3 at all points within the system. This correlated with lower TCC and ICC values within the DWDS. A small increase in ICC was observed within SR 4.1 and 4.2 during spring and summer, in which the percentage of intact cells increased from 13.49 to 24.61%. DWDS 4 (chloraminated) exhibited the smallest decline (21.97%) in chlorine concentration from post treatment to SR 4.3 (21.97%).

## Discussion

### AOC laboratory trials

This study developed a novel AOC methodology which combined the use of two known strains of bacteria, a larger inoculum volume and flow cytometric enumeration to increase the speed and reproducibility of AOC concentration. By using P-17 and NOX instead of a natural microbial inoculum, there is greater consistency between measurements. Unlike using known strains, the diversity of a natural inoculum was shown to depend upon the location and time in which the sample is taken. Hammes and Egli [21] use a fixed yield value of 1 x 10^7^ bacteria (μg of AOC) to convert the maximum growth of the natural microbial consortium to an AOC value, but this does not accurately reflect the changing bacterial composition of each water sample. This can therefore create difficulties in standardising the AOC method. In contrast, using P-17 and NOX facilitated robust replication and repeatability.

The analysis presented herein showed that increasing the density of P-17 and NOX decreased the time taken for the bacteria to reach stationary phase, whilst ensuring the growth yield values will be unaffected. Conversely, temperature changes decreased the peak in the bacterial growth rate, therefore requiring a different growth yield to be utilised. As the aim of this study was to create a standardised AOC it was deemed more suitable to use standardised yield values.

Comparison of enumeration methods used in the AOC test, highlighted that flow cytometry had equal or better reproducibility between triplicate measurements than standard plate counts, and therefore offers a suitable alternative to HPC’s in the AOC test. This is possibly a result of the selectivity of HPCs only culturing <1% of the population. Conversely, flow cytometry counts all cells present in the sample. The time and resources required to complete the assay were reduced significantly by using flow cytometric enumeration of the water samples, which removes the need for incubation of standard plate counts, hence is a more rapid method (2 minutes per sample compared to 3-4 days for HPC). Moreover, flow cytometry can detect a greater number of cells per sample (6 × 10^4^ events per analysis (the total volume analysed is 200 μL) before sample dilution is required [21]. It is only possible to detect up to 300 CFU per plate when using HPC. It should be noted that enumeration using flow cytometry required inoculation of bacterial strains P-17 and NOX into separate vials. As P-17 and NOX can consume some of the same organic substances [27, 28, 29], this can potentially lead to an overestimation of the AOC concentration. However, the AOC method used in this study produced AOC concentrations comparable to methods which inoculated P-17 and NOX into the same vial. Therefore, the substantial reduction in the time required to complete the method, as a result of using flow cytometric enumeration, was deemed justified. By combining the reproducibility of using two strains of bacteria with known growth rates, together with an increased inoculum density and cell enumeration using flow cytometry, this study presents a novel, fast and standardised AOC method suitable for application within urban water systems.

### AOC within WTW

The AOC test developed in this study was able to accurately capture fluctuations in AOC concentration entering and leaving the WTW, providing fast, repeatable results. The AOC concentration in treated water was predominantly influenced by source water quality. Groundwater systems contained a much lower concentration of AOC and produced treated water that could be classed as bio-stable if the AOC concentration was <100 μg/L. The final water AOC concentration at the majority of the treatment works supplied by surface water sources was found to contain AOC concentrations that exceed the existing criteria for bio-stable drinking water [9]. In general, drinking water containing a disinfectant is classed as biologically stable when it contains AOC concentrations of <100 μg/L with appropriate level of chlorine residual [16]. Taking this into account, the majority of the treated waters sampled are not classed as biologically stable.

Assimilable organic carbon is made up of small molecular weight particles, which are removed to different extents by different treatment processes. In this study an average of 40% AOC removal was found. Greatest AOC removal was achieved at WTW’s using rapid gravity filtration or granular activated carbon (GAC). GAC or biological activated carbon (BAC) is able to remove small easily biodegradable compounds. Easton and Jago [30] found coagulation and sedimentation (clarification) was able to achieve good removals (up to 57%) of AOC. The degree of AOC removal via membrane processes is dependent on the type of membrane. However, Escobar and Randall [31] found that Nanofiltration had no effect on AOC removal.

### AOC within DWDS

The AOC concentration DWDS was found to be a function of several factors including source water quality, chlorine residue, seasonality, AOC concentration removal at the treatment works and bacterial activity. As this study employs both SR inlet and outlet sampling, it is possible to identify changes in AOC concentration during hydraulic retention time within both piped sections and the SRs. When comparing the AOC concentration in post-treated water to SR outlet water, AOC was found to decline with increasing hydraulic residence time within the majority (7 out of 12 in both summer and winter) of DWDS, indicating that these systems were acting as net sinks of AOC. The decline in AOC in DWDS 2 and 4 correlated with an increase in ICC. A decrease in AOC with distance in the distribution systems has been found to correlate with an increase in heterotrophic bacteria [4, 8, 32].

To determine if the reduction of AOC levels in distribution may be caused by planktonic bacteria or those growing on the walls of the mains or SRs as biofilms, the AOC concentration in post treated water was compared to SR inlet water. The 12 DWDS consisted of 6 DWDS which are pipe only and 6 which pass through SR before reaching the final sampled SR (see Fig 1). The AOC concentration was found to increase within all of the pipe only sections during winter and 83% of pipe only sections during summer. In cases where organic matter is released from the biofilm, this can increase AOC and cell counts within the bulk water [32]. In contrast, the AOC concentration was found to decrease within 8 of the 12 SR sampled in both summer and winter. A reduction in AOC during elevated storage within a SR points to AOC consumption by heterotrophic bacteria.

The AOC concentration with DWDS is not only influenced by the degree of cell growth but also the disinfection residual. In DWDS 2, an initial increase in AOC concentration was observed before declining with distance in the network. The initial increase in AOC is a potential result of organic matter reacting with chlorine. One reason for an increase in AOC concentration is the oxidation of organic matter macromolecules into small-molecule biodegradable organic compounds (such as carboxylic acids) by chlorine or chloramine [33, 34]. Hence, the trade-off between achieving effective disinfection and limiting unwanted by-product formation (in this case AOC) becomes a main operational goal for water utilities. Despite AOC increasing after leaving the works, the presence of chlorine resulted in a low number of intact cells a short distance from the WTW. However, as total chlorine declines in the system from 0.95 mg/L to 0.53 mg/L at the outlet of SR 3, there is an increase in the number of intact cells with the percentage of ICC rising from 2% in post-treated water to >63% in SR2.

A seasonal trend was identified in DWDS 2-4, in which the AOC concentration generally increased to a maximum in spring, before declining from in summer and autumn. Polanska et al [35] also observed an increasing trend in the AOC concentration during spring. Summer regrowth was evident with DWDS 2 and 4, with an increase ICC within all SRs within DWDS 2 during summer. We conclude that a decrease in AOC from spring to summer is due to the consumption of AOC by heterotrophic organisms. This is demonstrated by an increase in the number of intact cells during summer. Similarly, there are fewest TCC and ICC within the winter months. The lower temperatures in winter would limit the bacterial regrowth during distribution and therefore AOC consumption by bacteria would be lower. It would also be likely that the reaction between chlorine and organic matter would also slow down at lower temperatures [36]. The combined effect of supressed microbial growth and slower oxidation of organic carbon, resulted in a continuous increase in AOC concentration during the winter season.

In DWDS 1 AOC values remained low (<125 μg/L) in post-treated water and within the DWDS. This site is supplied by borehole water containing low concentration of AOC. This highlights the importance of good source water quality. Similarly, DWDS 4 was found to contain AOC concentrations lower than DWDS 2 and 3 throughout distribution. Intact cell counts remained relatively low within DWDS 4 most likely as the AOC in the system was unable to support any additional regrowth. Organic carbon, especially AOC has been identified as a primary factor controlling microbial growth in drinking water distribution systems [37, 38]. DWDS 1 and DWDS 4 are also the two systems with the shortest hydraulic residence time (105 hrs in DWDS 1, 268 hrs in DWDS 4, 371 hrs in DWDS 2, 510 hrs in DWDS 3) (Fig 1).

In DWDS 3, supplied by chloraminated surface water, the AOC concentration was characterised by the highest AOC values and highest TCC. Despite having >40,000 TCC (cells/mL) within each SR, <9% of these were intact. It is likely that these cells attributed to the high AOC values with the system. The low proportion of intact cells within DWDS 3 also indicates efficient disinfection. The data suggests that chloramination is able to suppress the presence of intact cells more efficiently than free chlorine [39, 40]. This finding is attributed to the fact that chloramines are more effective than free chlorine for controlling biofilm regrowth in piped systems that is a major source for HPCs [41].

## Conclusions

The AOC assay developed in this study incorporates known strains P-17 and NOX, a higher inoculum volume and enumeration using flow cytometry to generate a quicker, robust method. By utilising known bacterial strains it is possible to use standardised yield curves to convert the cell count to AOC concentration. First application of the developed assay has confirmed its suitability for use in the field, capturing an extensive range of AOC loading in raw and post-treated water at 20 WTW. Subsequent analysis of AOC within 4 DWDS provided evidence of different pipe and service reservoir behaviour. The AOC concentration was found to increase within pipe only systems, whereas AOC declined within the SR. The results from this study highlight the complex cycling of AOC within DWDS, being a combination of both planktonic and biofilm processes within the pipes and SR. AOC was demonstrated to be a useful tool in assessing water quality and gaining understanding of DWDS behaviour.

## Supporting information

S1 Fig Fractions of carbon found within drinking water, including their universal acronyms. AOC (dashed box) is investigated in this study. (TIF)

S1 Table AOC methodologies incorporating either known strains of bacteria or a natural microbial inoculum. (DOCX)

S2 Table Methods used to measure general water quality parameters. (DOCX)

S3 Table Water temperature within post-treated water and each sampled service reservoir (SR) within drinking water distribution system (DWDS) (1-4). (DOCX)
