# Supplementary figures and images for: Application of enhanced assimilable organic carbon method across operational drinking water systems
Source: PLoS One. 2019 Dec 6;14(12):e0225477. doi: 10.1371/journal.pone.0225477 (PMC6897430; doi:10.1371/journal.pone.0225477)

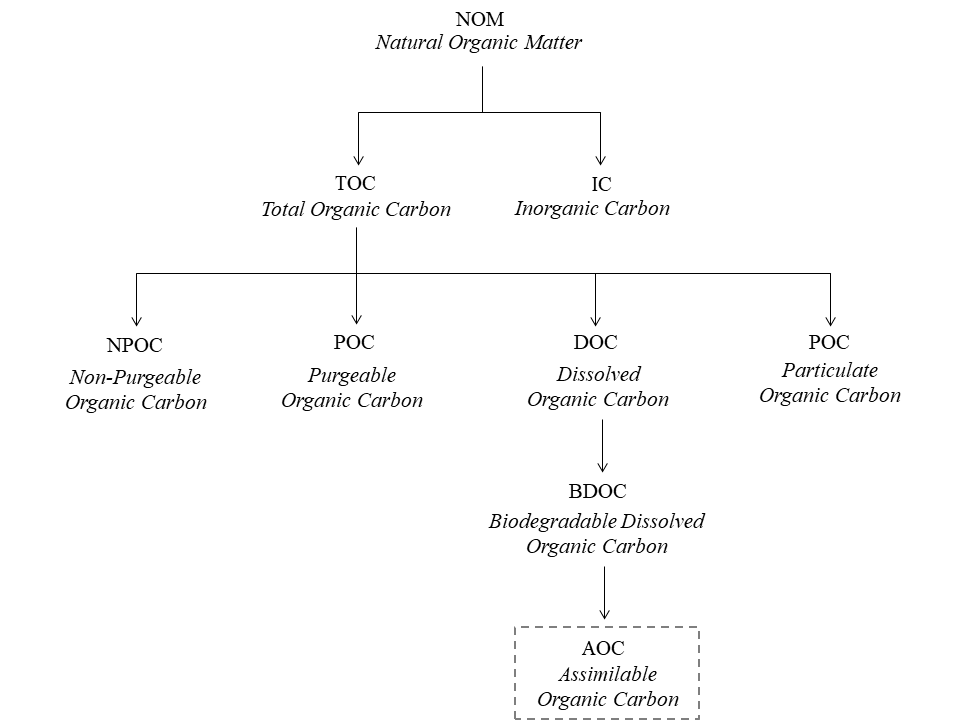

Supplement: S1 Fig — (TIF) [file pone.0225477.s001.tif]
