# Supplementary material for: Application of enhanced assimilable organic carbon method across operational drinking water systems
Source: PLoS One. 2019 Dec 6;14(12):e0225477. doi: 10.1371/journal.pone.0225477 (PMC6897430; doi:10.1371/journal.pone.0225477)
Supplement: S1 Table — (DOCX) [file pone.0225477.s002.docx]

**Table 1: AOC methodologies incorporating either known strains of bacteria or a natural microbial inoculum.**

| **Inoculum Type** | **Sample Preparation** | **Inoculum** | **Incubation** | **Enumeration** | **Yield Factors** | **Reference** |
| --- | --- | --- | --- | --- | --- | --- |
| Known strains of bacteria | Pasteurisation 60 °C for 30 mins | 500 CFU/mL P-17 only | 15 °C no shaking | HPC 25 °C 40-48 hours | 4.1x10^6^ P-17 | Van der Kooij et al [1] |
|  |  | 100-300 CFU/mL of strains P-17 and NOX |  |  | 4.1 x 10^6^ P-17, 1.2 x 10^7^ NOX | Van der Kooij [2] |
|  |  | Either strain P-17 or strain NOX | Room temp for 1-3 days | ATP | * | LeChevallier et al [3] |
|  | Pasteurisation 70 °C for 1 hour |  | Room temp 3-5 days | HPC 25 °C for 7 days | 4.1 x 10^6^ P-17, 1.2 x 10^7^ NOX | Escobar & Randall [4] |
|  | Pasteurisation 70 °C for 30 min |  |  |  |  | Escobar & Randall [5] |
|  |  | Inoculate with P-17, incubate & enumerate, pasteurise again, inoculate NOX, incubate & enumerate | 25 °C for 2 days for P-17 and 3 days for NOX | HPC 25 °C 3 days |  | Liu et al [6] |
|  | Pasteurisation 70 °C for 30 min followed by 15 min at 70 °C in heating cabinet | P-17, PF-1 or a combined inoculum of P17/NOX to final concentration 50–500 CFU/mL | 20 °C with shaking |  | * | Charnock and Kjønnø [7] |
|  | Pasteurisation 30 min at 60 °C, rapidly cooled in ice bath to 15 °C | Inoculated with P17 and NOX simultaneously, to reach 300 CFU/mL of each strain | 15 °C | HPC every 2 days | 4.6 x 10^6^ P-17, 1.3 x 10^7^ NOX | Polanksa et al [8] |
|  | Pasteurisation 70 °C for 30 min | 500 CFU/mL each of P-17 and NOX |  | 25 °C for 3 to 5 days | 4.1 x 10^6^ P-17, 1.2 x 10^7^ NOX | APHA [9] |
|  | Samples filtered 0.3 μm filter, pasteurisation 70 °C for 30 min |  |  | HPC R2A agar 7, 8, and 9 days |  | Thayanukul et al [10] |
|  | Pasteurisation 70 °C for 40 min | P-17 and NOX | 25 °C 1-8 days | HPC on nutrient agar | * | Lou et al [11]; Han et al [12];  Lou et al [13] |
|  | Pasteurisation 75 °C for 30 min | Samples inoculated simultaneously with P-17 and NOX | 20 °C | Pour plating on R2A agar 14–20 days at 20 °C | 4.53 x 10^6^ P-17, 1.56 x 10^7^ NOX | Ohkouchi et al [14]; Ohkouchi et al [15] |
|  | * | * | 22 °C–25 °C | HPC 1–5 days 25 °C | * | Zhao et al [16] |
|  | Pasteurisation 70 °C for 30 min | 1 mL of either P17 or NOX | 23 °C for 5 days | Flow Cytometry | 4.33 x 10^6^ P-17, 1.03 x 10^7^ | Aggarwal et al [17] |
|  |  |  |  |  |  |  |
| Natural microbial inoculum | 1 x 10^7^ cells µg C/L | Filtered 0.22 µm (not pasteurised) | Triplicate vials inoculated with 100 µL giving a final concentration of 5x 10^3^ (±2 x 10^2^) cells mL^-1^ | 30 °C until stationary phase | Flow cytometry | Hammes and Egli [18] |
|  |  |  | Cell concentration of 1 x 10^4^ cell / mL |  |  | Hammes et al [19] |
|  |  |  |  |  |  | Hammes and Egli [20] |
|  |  |  |  |  |  | Bazri & Mohseni [21] |
|  |  | Autoclaved (20 min 12 °C), filtered 0.22 µm |  |  |  | Vital et al [22] |
|  |  | Sample pasteurised, filtered 0.22 µm |  |  |  | Liu et al [23]; Vital et al [24];  Hammes et al [25] |
|  |  | Filtered 0.2 μm, pasteurised 60 °C for 30 min |  |  |  | Elhadidy et al [26] |
|  | * | * | * | * | Turbidity (NTU) | Hambsch and Werner [27]; Werner and Hambsch [28] |

Details not included in reference. NOX = *Spirillum* strain NOX; P-17 = *Pseudomonas fluorescens* strain P-17; PF-1 = isolate of *Pseudomonas fluorescens*, ATP = Adenosine Triphosphate; R2A = Reasoner's 2A agar; HPC = heterotrophic plate count; C = carbon, NTU = Nephelometric Turbidity Unit.

1. Van der Kooij D, Visser A, Hijnen W. Determining the concentration of easily assimilable organic carbon in drinking water. American Water Works Association. 1982; 74(10): 540–543.
2. Van der Kooij D. Assimilable organic carbon as an indicator of bacterial regrowth. American Water Works Association. 1992; 57–65.
3. LeChevallier MW, Shaw NE, Kaplan LA, Bott TL. Development of a rapid assimilable organic carbon method for water. Applied and Environmental Microbiology. 1993; 59(5): 1526–1531.
4. Escobar IC, Randall AA. Influence of nanofiltration on distribution system biostability. American Water Works Association. 1999; 91(6): 76-89.
5. Escobar IC, Randall AA. Sample storage impact on the assimilable organic carbon (AOC) bioassay. Water research. 2000; 34(5); 1680-1686.
6. Liu W, Wu H, Wang Z, Ong SL, Hu JY, Ng WJ. Investigation of assimilable organic carbon (AOC) and bacterial regrowth in drinking water distribution system. Water Research. 2002; 36: 891–898.
7. Charnock C, Kjønnø O. Assimilable organic carbon and biodegradable dissolved organic carbon in Norwegian raw and drinking waters. Water Research. 2000; 34(10): 2629-2642.
8. Polanska M, Huysman K, Van Keer C. Investigation of assimilable organic carbon (AOC) in Flemish drinking water. Water Research. 2005; 39(11): 2259-2266.
9. American Public Health Association (APHA). Standard Methods for the Examination of Water and Wastewater, 21st Edition, American Public Health Association, New York; 2005.
10. Thayanukul P, Kurisu F, Kasuga I, Furumai H. Evaluation of microbial regrowth potential by assimilable organic carbon in various reclaimed water and distribution systems. Water Research. 2013; 47(1): 225-232.
11. Lou JC, Yang CY, Chang CJ, Chen WH, Tseng WB, Han JY. Analysis and removal of assimilable organic carbon (AOC) from treated drinking water using a biological activated carbon filter system. Journal of Environmental Chemical Engineering. 2014; 2(3): 1684-1690.
12. Han JY, Kai-Lin H, Chung-Yi L, Lou JC, Wu MC. Control of assimilable organic carbon (AOC) concentrations in a water distribution system. Desalination and Water Treatment. 2012; 47(1-3): 11-16.
13. Lou JC, Chen BH, Chang TW, Yang HW, Han JY. Variation and removal efficiency of assimilable organic carbon (AOC) in an advanced water treatment system. Environmental Monitoring and Assessment. 2011; 178(1): 73-83.
14. Ohkouchi Y, Ly BT, Ishikawa S, Kawano Y, Itoh S. Determination of an acceptable assimilable organic carbon (AOC) level for biological stability in water distribution systems with minimized chlorine residual. Environmental Monitoring and Assessment. 2013; 185(2): 1427–1436.
15. Ohkouchi Y, Ly BT, Ishikawa S, Aoki Y, Echigo S, Itoh S. A survey on levels and seasonal changes of assimilable organic carbon (AOC) and its precursors in drinking water. Environmental Technology. 2011; 32(14): 1605-1613.
16. Zhao X, Hu H, Liu S, Jiang F, Shi X, Li M. Improvement of the assimilable organic carbon (AOC) analytical method for reclaimed water. Frontiers of Environmental Science & Engineering. 2013; 7(4): 483-491.
17. Aggarwal S, Jeon Y, Hozalski RM. Feasibility of using a particle counter or flow-cytometer for bacterial enumeration in the assimilable organic carbon (AOC) analysis method. Biodegradation. 2015; 26(5): 387-397.
18. Hammes FA, Egli T. ‘New method for assimilable organic carbon determination using ﬂow-cytometric enumeration and a natural microbial consortium as inoculum’, Environmental Science and Technology. 2005; 39(9): 3289–3294.
19. Hammes F, Salhi E, Köster O, Kaiser HP, Egli T, Von Gunten U. Mechanistic and kinetic evaluation of organic disinfection by-product and assimilable organic carbon (AOC) formation during the ozonation of drinking water. Water Research. 2006; 40(12): 2275-2286.
20. Hammes FA, Egli T. A flow cytometric method for AOC determination. TECHNEU. 2007.
21. Bazri MM, Mohseni M. A rapid technique for assessing assimilable organic carbon of UV/H2O2-treated water. Journal of Environmental Science and Health. 2013; 48: 1086–1093.
22. Vital M, Füchslin HP, Hammes F, Egli T. Growth of Vibrio cholerae O1 Ogawa Eltor in freshwater. Microbiology. 2007; 153(7): 1993-2001.
23. Liu X, Wang J, Liu T, Kong W, He X, Jin Y, et al. Effects of Assimilable Organic Carbon and Free Chlorine on Bacterial Growth in Drinking Water. PLoS ONE. 2015; 10(6): e0128825. doi:10.1371/ journal.pone.0128825.
24. Vital M, Dignum M, Magic-Knezev A, Ross P, Rietveld L, Hammes F. Flow cytometry and adenosine tri-phosphate analysis: Alternative possibilities to evaluate major bacteriological changes in drinking water treatment and distribution systems. Water Research. 2012; 46: 4665-4676.
25. Hammes F, Berger C, Köster O, Egli T. Assessing biological stability of drinking water without disinfectant residuals in a full-scale water supply system. Journal of Water Supply: Research and Technology-AQUA. 2010; 59(1): 31-40.
26. Elhadidy AM, Van Dyke MI, Peldszus S, Huck PM. Application of flow cytometry to monitor assimilable organic carbon (AOC) and microbial community changes in water. Journal of Microbiological Methods. 2016: 130; 154–163.
27. Hambsch B, Werner P. In automated measurement of bacterial growth curves for the characterization of organic substances in water. Water Quality and Technology Conference, San Diego, USA; 1990.
28. Werner P, Hambsch B. Investigations on the growth of bacteria in drinking water. Water Supply. 1986; 4(3): 227-232.
