# Supplementary material for: Application of enhanced assimilable organic carbon method across operational drinking water systems
Source: PLoS One. 2019 Dec 6;14(12):e0225477. doi: 10.1371/journal.pone.0225477 (PMC6897430; doi:10.1371/journal.pone.0225477)
Supplement: S2 Table — (DOCX) [file pone.0225477.s003.docx]

| **Parameter** | **Method** | **Reference** |
| --- | --- | --- |
| **Total (TCC) and Intact Cell Counts (ICC) (Cells/mL)** | 500 µl samples were stained with 5 µl of SYBR Green (Life Sciences) for TCC. 500 µl samples were stained with 6 µl volume of SYBR Green/ Propidium Iodine mixture (Life Sciences) (with a final concentration of 1x SYBR Green and 3μM PI) for ICC. Samples were analysed using a BD Accuri C6 Flow Cytometer with autosampler (BD Accuri, UK). The proportion of intact cell counts (%) is defined as intact cell count / total cell count * 100. | Gillespie et al [1] |
| **ATP** | ATP measurements were made using the BacTiter-GloTM Microbial Cell Viability Assay (Promega, UK). Total ATP was established using the BacTiter-Glo reagent (G8231; Promega Corporation) and a luminometer (Tecan). | Promega Corporation [2] |
| **Free and total chlorine (mg/L)** | The measurement of free and total chlorine residuals was performed at the time of sampling, using a pocket colorimeter (Hach-Lange, Salford, U.K.). | Gillespie et al [1] |
| **Heterotrophic plate counts (CFU/mL)** | Heterotrophic plate counts of drinking water samples were determined by mixing 1 mL of sample into 18 mL of molten YEA (Yeast Extract Agar, cat. No. CM0019B, Oxoid Ltd., ThermoFisher Scientific, Loughborough, U.K.). Plates were incubated at 37 °C for 48 h or at 22 °C for 72 h. | Gillespie et al [1] |

# References

1. Gillespie S, Lipphaus P, Green J, Parsons S, Weir P, Juskowiak K, et al. Assessing microbiological water quality in drinking water distribution systems with disinfectant residual using flow cytometry. Water Research. 2014; 65: 224-234.
2. Promega Corporation. Technical Bulletin: BacTiter-Glo™ Microbial Cell Viability Assay. 2016. www.promega.com/protocols/.
