# Supplementary material for: Application of enhanced assimilable organic carbon method across operational drinking water systems
Source: PLoS One. 2019 Dec 6;14(12):e0225477. doi: 10.1371/journal.pone.0225477 (PMC6897430; doi:10.1371/journal.pone.0225477)
Supplement: S3 Table — (DOCX) [file pone.0225477.s004.docx]

| DWDS | Location in DWDS | Spring Water Temperature | Summer Water Temperature | Autumn Water Temperature | Winter Water Temperature |
| --- | --- | --- | --- | --- | --- |
| 1 | Post-treated | 9.1 | 9.7 | 9.2 | 8.2 |
|  | SR 1.1 | 8.8 | 10.0 | 8.6 | 7.6 |
|  | SR 1.2 | 8.1 | 11.1 | 8.4 | 5.8 |
|  | SR1.3 | 8.1 | 11.8 | 8.6 | 5.7 |
| 2 | Post-treated | 10.2 | 16.0 | 8.1 | 4.0 |
|  | SR 2.1 | 10.0 | 15.6 | 8.7 | 4.8 |
|  | SR 2.2 | 9.2 | 14.2 | 10.4 | 5.9 |
|  | SR 2.3 | 9.3 | 15.0 | 8.8 | 5.5 |
| 3 | Post-treated | 10.9 | 16.5 | 7.2 | 3.8 |
|  | SR 3.1 | 9.7 | 15.1 | 8.1 | 4.4 |
|  | SR 3.2 | 9.5 | 15.2 | 9.0 | 5.0 |
|  | SR 3.3 | 7.8 | 12.8 | 9.5 | 5.1 |
| 4 | Post-treated | 10.9 | 16.5 | 7.3 | 4.3 |
|  | SR 4.1 | 9.6 | 15.5 | 6.9 | 4.2 |
|  | SR 4.2 | 8.5 | 13.7 | 7.0 | 5.3 |
|  | SR 4.3 | 8.4 | 13.2 | 8.9 | 5.1 |
